# Precision in Liver Diagnosis: Varied Accuracy Across Subgroups and the Need for Variable Thresholds in Diagnosis of MASLD

**DOI:** 10.1111/liv.16240

**Published:** 2025-01-24

**Authors:** Yasaman Vali, Anne‐Marieke van Dijk, Jenny Lee, Jerome Boursier, Vlad Ratziu, Carla Yunis, Jörn M. Schattenberg, Luca Valenti, Manuel Romero Gomez, Detlef Schuppan, Salvatore Petta, Mike Allison, Mark L. Hartman, Kimmo Porthan, Jean‐Francois Dufour, Elisabetta Bugianesi, Amalia Gastadelli, Zoltan Derdak, Celine Fournier‐Poizat, Elizabeth Shumbayawonda, Michael Kalutkiewicz, Hannele Yki‐Jarvinen, Mattias Ekstedt, Andreas Geier, Aldo Trylesinski, Sven Francque, Clifford Brass, Michael Pavlides, Adriaan G. Holleboom, Max Nieuwdorp, Quentin M. Anstee, Patrick M. Bossuyt, the LITMUS investigators

**Affiliations:** ^1^ Department of Epidemiology and Data Science Amsterdam University Medical Centres Amsterdam The Netherlands; ^2^ Amsterdam Gastroenterology Endocrinology Metabolism (AGEM) Institute, Amsterdam UMC University of Amsterdam Amsterdam The Netherlands; ^3^ Department of Internal and Vascular Medicine Amsterdam University Medical Centres Amsterdam The Netherlands; ^4^ Laboratoire HIFIH, UPRES EA 3859, SFR ICAT 4208 Université d'Angers Angers France; ^5^ Service d'Hépato‐Gastroentérologie et Oncologie Digestive Centre Hospitalier Universitaire d'Angers Angers France; ^6^ Assistance Publique‐Hôpitaux de Paris, Hôpital Pitié Salpêtrière ICAN (Institute of Cardiometabolism and Nutrition), Sorbonne University Paris France; ^7^ Pfizer Research and Development, Pfizer Inc Lake Mary Florida USA; ^8^ Department of Internal Medine II Saarland University Medical Center Homburg Germany; ^9^ Saarland University Saarbrücken Germany; ^10^ Department of Pathophysiology and Transplantation Università Degli Studi di Milano Milano Italy; ^11^ Precision Medicine Biological Resource Center Unit, Fondazione IRCCS Ca' Granda Policlinico Milano Italy; ^12^ Digestive Diseases Unit Hospital Universitario Virgen del Rocío Sevilla Spain; ^13^ Hepatic and Digestive Diseases Networking Biomedical Research Centre (CIBERehd) Instituto de Biomedicina de Sevilla Sevilla Spain; ^14^ Universidad de Sevilla Sevilla Spain; ^15^ Institute of Translational Immunology University Medical Center Mainz Mainz Germany; ^16^ Division of Gastroenterology Beth Israel Deaconess Medical Center Boston Massachusetts USA; ^17^ Sezione di Gastroenterologia e Epatologia, PROMONISE Department Università di Palermo Palermo Italy; ^18^ Liver Unit, Department of Medicine, Cambridge NIHR Biomedical Research Centre Cambridge University NHS Foundation Trust Cambridge UK; ^19^ Lilly Research Laboratories Eli Lilly and Company Indianapolis Indiana USA; ^20^ Department of Medicine University of Helsinki and Helsinki University Hospital Helsinki Finland; ^21^ Minerva Foundation Institute for Medical Research Helsinki Finland; ^22^ Centre Des Maladies Digestives Lausanne Switzerland; ^23^ Department of Medical Sciences, Division of Gastro‐Hepatology, A.O. Città della Salute e della Scienza di Torino University of Turin Turin Italy; ^24^ Institute of Clinical Physiology National Research Council Pisa Italy; ^25^ GI DDU, Takeda Pharmaceuticals Company Ltd. Cambridge Massachusetts USA; ^26^ Echosens Paris France; ^27^ Perspectum Oxford UK; ^28^ Resoundant Rochester Minnesota USA; ^29^ Department of Health, Medicine and Caring Sciences Linköping University Linköping Sweden; ^30^ Division of Hepatology, Department Medicine II Wurzburg University Hospital Wurzburg Germany; ^31^ Advanz Pharma Capital House London UK; ^32^ Department of Gastroenterology Hepatology, and Laboratory of Experimental Medicine and Paediatrics, Antwerp University Hospital University of Antwerp Antwerp Belgium; ^33^ Novartis Pharmaceuticals Corporation East Hanover New Jersey USA; ^34^ Radcliffe Department of Medicine and Oxford NIHR Biomedical Research Centre University of Oxford Oxford UK; ^35^ Translational and Clinical Research Institute, Faculty of Medical Sciences Newcastle University Newcastle upon Tyne UK; ^36^ Newcastle NIHR Biomedical Research Centre Newcastle upon Tyne Hospitals NHS Trust Newcastle upon Tyne UK

**Keywords:** non‐alcoholic liver disease, non‐alcoholic steatohepatitis, non‐invasive liver tests, subgroup analysis

## Abstract

**Background and Aims:**

The performance of non‐invasive liver tests (NITs) is known to vary across settings and subgroups. We systematically evaluated whether the performance of three NITs in detecting advanced fibrosis in patients with metabolic dysfunction‐associated steatotic liver disease (MASLD) varies with age, sex, body mass index (BMI), type 2 diabetes mellitus (T2DM) status or liver enzymes.

**Methods:**

Data from 586 adult LITMUS Metacohort participants with histologically characterised MASLD were included. The diagnostic performance of the Fibrosis‐4 Index (FIB‐4), enhanced liver fibrosis (ELF) and vibration‐controlled transient elastography liver stiffness measurement (VCTE LSM) was evaluated. Performance was expressed as the area under the receiver operating characteristics curve (AUC). Thresholds for detecting advanced fibrosis (≥F3) were calculated for each NIT for fixed (high) sensitivity, specificity and predictive values.

**Results:**

Differences in AUC between all subgroups were small and statistically not significant, indicating comparable performance in detecting ≥F3, irrespective of these clinical factors. However, different thresholds were needed to achieve the same level of accuracy with each test. For example, for a fixed sensitivity and specificity, the thresholds for all three NITs were higher in patients with T2DM. Effects for sex, age and liver enzymes were less pronounced.

**Conclusions:**

Performance of the selected NITs in detecting advanced liver fibrosis does not vary substantially with clinical characteristics. However, different thresholds have to be selected to achieve the same sensitivity, specificity and predictive values in the respective subgroups. Large prospective studies are called for to study NIT accuracy considering multiple patient characteristics.

**Abbreviations**
95% CI: 95% confidence interval
ALT: alanine aminotransferase
AST: aspartate aminotransferase
AUC: area under each ROC curve
BMI: body mass index
ELF: enhanced liver fibrosis test
FIB‐4: fibrosis‐4 index
GGT: gamma‐glutamyl transferase
MASLD: metabolic dysfunction‐associated steatotic liver disease
MASH: metabolic dysfunction‐associated steatohepatitis
NAFLD: non‐alcoholic fatty liver disease
NASH: non‐alcoholic steatohepatitis
NITs: non‐invasive tests
PPV/NPV: positive and negative predictive values
ROC: receiver operating characteristic
TG: triglyceride
T2DM: type 2 diabetes mellitus
VCTE: vibration‐controlled transient elastography

**Summary**

- Non‐invasive tests are used to check for severe liver damage in people with fatty liver, but their accuracy may vary depending on patient characteristics.
- This study found that threshold values of non‐invasive tests need to be adjusted for different groups based on their age, gender, weight or diabetes status.
- This means that tailoring the tests' thresholds is essential to ensure they provide accurate results for everyone.

## 1 Introduction

In parallel with the global rise of obesity and type 2 diabetes mellitus (T2DM), the worldwide prevalence of metabolic dysfunction‐associated steatotic liver disease (MASLD), previously known as non‐alcoholic fatty liver disease (NAFLD), is increasing [1, 2]. MASLD represents a conceptual shift from the previous terminology of NAFLD, indicating a better understanding of the disease's complexity and the connection between metabolic dysregulation and liver pathology. This disorder poses a significant public health challenge, given its association with obesity, insulin resistance and cardiovascular risk factors. As MASLD can progress and lead to serious conditions, accurate and non‐invasive tests (NITs) are crucial for timely intervention and effective management [3, 4].

The current reference standard for detecting metabolic dysfunction‐associated steatohepatitis (MASH) and staging liver fibrosis in patients with MASLD is liver biopsy. Due to the invasive and resource‐intensive nature and a small but noticeable risk of complications of this procedure [5], particular attention has been given to the development of NITs for MASLD in recent years [6].

Several NITs for detecting MASH and fibrosis have been reported in the literature. Some are recommended by guidelines in clinical care path development for patients with MASLD‐MASH [6]. Examples are the Fibrosis‐4 Index (FIB‐4), a simple fibrosis index relying on routinely measured clinical (chemistry) markers [7, 8], the Enhanced Liver Fibrosis test (ELF), a patented panel of three direct serum markers related to connective tissue turnover [9] and liver stiffness measurement by vibration‐controlled transient elastography (VCTE LSM) using the FibroScan device [6, 10].

Despite their mention in guidelines, variations in the reported performance of these NITs raise concerns about their reliability and potential impact on accurate diagnosis and effective disease management [11, 12, 13, 14, 15, 16, 17]. It is possible that the variability in performance is due, at least in part, to identifiable differences between study groups and clinical settings. For instance, it is reported by a study that high BMI may affect the diagnostic accuracy of VCTE LSM, leading to either overestimation or underestimation of the stage of fibrosis, while another study showed that non‐obese patients with MASLD had lower VCTE LSM levels [18]. One study reported that at higher age, the specificity for FIB‐4 to stage advanced fibrosis may have been unacceptably low, [19] while another study showed significantly higher ELF results in healthy male controls compared to females [20].

This study addresses these concerns by exploring the variability in diagnostic accuracy of three liver NITs in detecting advanced fibrosis (fibrosis stage ≥ 3) across different clinical subgroups. Using a large sample of well‐defined subjects from the Liver Investigation: Testing Marker Utility in Steatohepatitis (LITMUS) metacohort data of the European NAFLD Registry [21], we explored subgroups defined by anthropometric and metabolic factors. Moreover, this study builds upon our recent publication of the LITMUS study [12, 15, 22], highlighting the importance of our research within the evolving field of MASLD research. By examining how well diagnostic tests perform in various MASLD patients, our intention was to present this study as a demonstration of foundational validity, focusing on individual features to highlight broader concerns about variations in test accuracy.

## 2 Methods

### 2.1 Participants (LITMUS Metacohort)

We evaluated 966 adult participants with biopsy‐proven MASLD from the LITMUS metacohort of the prospective European NAFLD Registry [21]. Patients were recruited from 13 countries across Europe between 2010 and 2019. All participants, initially recruited during two EU‐funded projects, EPoS [23] and FLIP [24], provided informed consent prior to inclusion. The contributing projects were approved by the relevant Ethical Committees in the participating countries, and studies were conducted according to the guidelines of the Declaration of Helsinki.

Eligible for this analysis were participants with paired liver biopsy and serum samples. All patients that met our inclusion criteria had undergone a liver biopsy as part of the routine diagnostic workup for presumed MASLD. Patients with excessive alcohol consumption (> 20–30 g/day), other chronic liver diseases, such as viral hepatitis B or C, and incomplete data for analysis were excluded.

### 2.2 Clinical Assessments

Clinical data on anthropometric factors, medical history and lifestyle were collected in the respective recruiting centres. The body mass index (BMI) was calculated as weight in kg over height in m^2^. Blood samples were also collected locally and assessed in local laboratories by routine clinical assays: Lipid (LDL, HDL, cholesterol, triglyceride (TG)) and liver profiles (platelet count, alanine aminotransferase (ALT), aspartate aminotransferase (AST) and gamma‐glutamyl transferase (GGT)). Patients were considered to have diabetes if they had a prior diagnosis of diabetes or had fasting glucose > 7.0 mmol/L.

### 2.3 Histology

Liver biopsy samples were collected in each recruiting centres and evaluated locally by expert liver pathologists in respective centres, prospectively following clinical work‐up [24]. The Non‐Alcoholic Steatohepatitis Clinical Research Network (NASH CRN) system was used for grading the disease activity: steatosis and lobular inflammation were scored from 0 to 3; ballooning from 0 to 2; and liver fibrosis was staged from 0 to 4, where advanced fibrosis was defined as F ≥ 3 [25].

### 2.4 Non‐Invasive Tests

All serum samples were collected in standardised collection kits, stored at −80°C, then shipped to Nordic Biosciences, a CLIA‐certified laboratory, and blinded to clinical data for analysis. The eligible sample‐liver biopsy interval was six months.

The three following NITs were evaluated.

- ELF test, with scores based on hyaluronic acid, tissue inhibitor of matrix metalloproteinase‐1 and aminoterminal propeptide of procollagen type III, measured in the Central Laboratory using the Siemens Advia Centaur device [9, 26].
- FIB‐4 index, with scores calculated using the available clinical laboratory data based on the following formula: age × AST (IU/L)/platelet count (×10^9^/L) × √ALT (IU/L) [7, 8].
- Liver stiffness, measured locally by (VCTE LSM) (FibroScan, Echosens, Paris, France) by centres [27]. Probe sizes were selected based on the device guidelines' advice.

### 2.5 Statistical Analyses

We first evaluated the performance of the tests based on current clinical practice and European Association for the Study of the Liver (EASL) [28] guidelines, using histology as the reference standard. The EASL guideline for managing patients with MASLD recommends a stepwise approach starting with blood‐based scores (e.g., FIB‐4) followed by imaging techniques (e.g., VCTE) to rule out advanced fibrosis. In our cohort, we assessed the performance of VCTE and the ELF test for this stepwise approach, applying the thresholds recommended by EASL.

Then we assessed the diagnostic performance of the three NITs in detecting advanced fibrosis (F ≥ 3) across different subgroups. Eligible patients were categorised in six sets of subgroups based on (1) their sex (female, male) (2); BMI (normal BMI < 25 kg/m^2^, overweight BMI 25–30 kg/m^2^ or obese BMI ≥ 30 kg/m^2^) (3); age (18–45, 45–65 and > 65 years) (4); diabetes status (T2DM or no T2DM) (5); AST (lower or greater than 40 (U/L)); and (6) ALT (lower or greater than 45 (U/L)) [19].

For each subgroup, we constructed non‐parametric, empirical receiver operating characteristic (ROC) curves and calculated the area under each ROC curve (AUC) with its 95% confidence interval (95% CI), using the DeLong method. DeLong's test was applied to evaluate if the difference between the AUC values of the subgroups is significant (*p* < 0.05) [29].

To reduce bias due to confounding from sex, age, BMI and T2DM status, we also estimated covariate‐adjusted AUCs for these variables [30]. For this, we used the ‘ROCnReg’ package in R and its AROC.bnp method, which implements a nonparametric Bayesian approach based on a single‐weights dependent Dirichlet process mixture of normal distributions and the Bayesian bootstrap [31].

We then defined thresholds of the respective NITs to detect advanced fibrosis at pre‐specified (high) sensitivity and specificity in each subgroup, based on the non‐parametric ROC curve.

Additionally, we specified desired positive and negative predictive values (PPV/NPV) to rule in or out advanced fibrosis, based on the recommended thresholds of the respective tests in the literature. We calculated thresholds for the NITs to achieve these pre‐specified PPV and NPV, while considering the proportion of participants with advanced fibrosis in each subgroup of patients as an expression of the prevalence. All statistical analyses were performed using R software version 4.0.5.

## 3 Results

### 3.1 Study Group Characteristics

Of the 966 Metacohort participants, 960 had data for all relevant clinical variables and at least one NIT. From those, 586 patients had all three non‐invasive test results available. In the 586 participants included in the analysis, the mean age was 52 years, mean BMI was 31 kg/m^2^, 64% were men and 44% had T2DM. Fibrosis: F0 (26%), F1 (21%), F2 (25%), F3 (21%) and F4 (7%). Patient characteristics are summarised in Tables 1, S2 and S3.

**TABLE 1:** Characteristics of the study group.

	Entire metacohort (*n* = 966)	Subgroup with all three tests and clinical data (*n* = 586)	Study group with missing biomarker or clinical data (*n* = 380)
Age, years	51.2 (13.0)	52.0 (13.2)	50.0 (12.6)
Female	403 (42%)	211 (36%)	191 (50%)
BMI, kg/m^2^	34.12 (8.26)	31.3 (6.02)	38.5 (9.11)
Diabetes	406 (42%)	257 (43.9%)	148 (39%)
ALT, U/L	62.7 (42.5)	66.3 (40.4)	57.0 (45.2)
AST, U/L	42.9 (26.0)	44.4 (25.7)	40.4 (26.3)
GGT, U/L	108 (155)	99.5 (111)	122 (207)
Albumin, g/L	4.38 (0.419)	4.38 (0.408)	4.39 (0.436)
Platelet count, 10^9^/L	239 (73.4)	236 (70.4)	245 (77.5)
Glucose, mmol/L	6.42 (2.47)	6.33 (2.25)	6.57 (2.80)
Triglycerides, mg/L	2.08 (1.18)	2.05 (1.09)	2.14 (1.31)
VCTE LSM probe size			
XL‐probe	130 (13.5%)	112 (19.1%)	17 (4.5%)
M‐probe	484 (51.0%)	452 (77.1%)	32 (8.4%)
Missing	352 (36.4%)	22 (3.4%)	331 (87.1%)
Liver fibrosis stage			
0	309 (32%)	152 (26%)	157 (41%)
1	186 (19%)	121 (21%)	65 (17%)
2	198 (21%)	148 (25%)	50 (13%)
3	188 (19%)	122 (21%)	66 (17%)
4	85 (9%)	43 (7%)	41 (11%)
NASH^a^	512 (53%)	323 (55%)	188 (50%)

*Note:* All continuous variables are expressed as mean (SD).

Abbreviations: ELF, enhanced liver fibrosis; FIB‐4, Fibrosis‐4 Index; NASH, non‐alcoholic steatohepatitis; VCTE LSM, vibration controlled transient elastography liver stiffness measurement.

^a^ NAS score of ≥ 4 with at least one point in each component (inflammation, ballooning, steatosis).

### 3.2 Performance of Tests in Stepwise Approach

**FIGURE 1:**
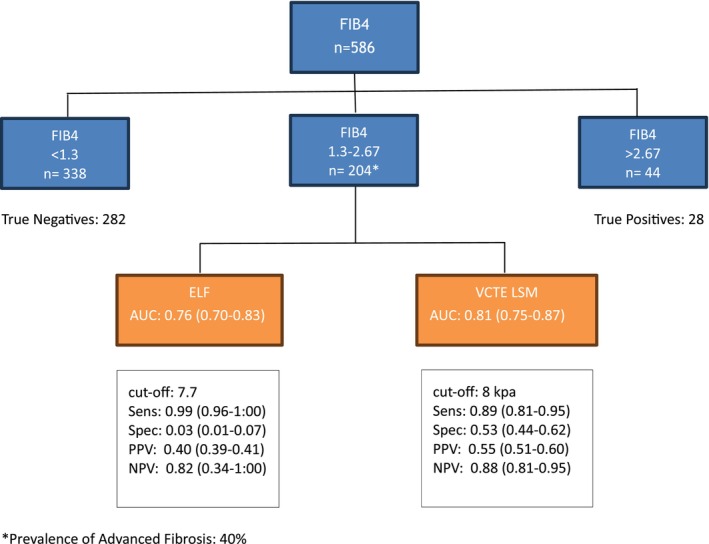
Stepwise strategy using FIB‐4, ELF, and VCTE for non‐invasive assessment of advanced fibrosis.

Using the EASL‐recommended stepwise approach, FIB‐4 stratified patients into three categories: low‐risk (< 1.3), indeterminate (1.3–2.67) and high‐risk (> 2.67) for advanced fibrosis. Of the 586 patients, 204 had an ‘indeterminate’ FIB‐4 result and could be evaluated for further assessment by a second test. In this group, ELF (AUC: 0.76, 95% CI: 0.70–0.83) and VCTE LSM (AUC: 0.81, 95% CI: 0.75–0.87) were evaluated using thresholds of 7.7 and 8 kPa to rule out advanced fibrosis, respectively. ELF showed a sensitivity of 99% and NPV of 82% while VCTE LSM had a sensitivity of 89% and NPV of 88% (Figure 1).

### 3.3 Overall Performance of NITs for Detecting F ≥ 3 in Different Subgroups

**FIGURE 2:**
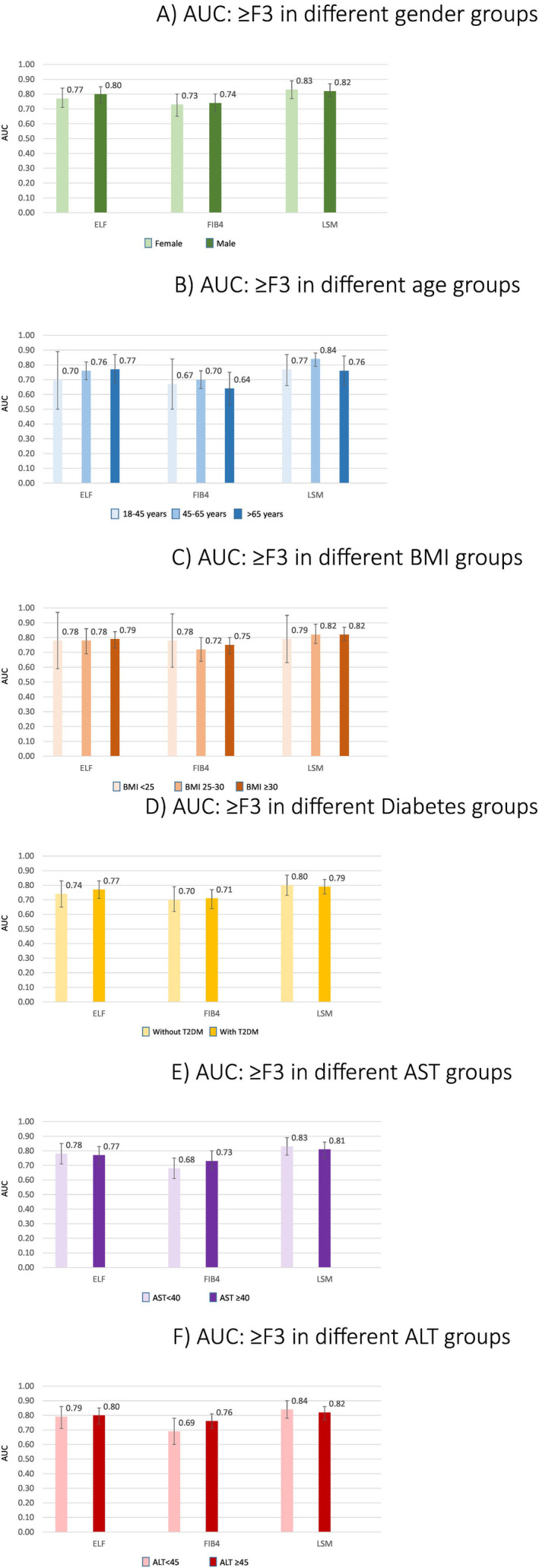
The performance (AUCs) of the tests for detecting advanced fibrosis in different. (A) Gender groups; females (*N* = 211) and males (*N* = 375), (B) age groups; 18–45 years. (*N* = 157); 45–65 years (*N* = 331); > 65 years (*N* = 98), (C) body mass index (BMI in kg/m^2^); BMI < 25 (*N* = 70); BMI 25–30 (*N* = 209); BMI ≥ 30 (*N* = 307), (D) diabetes status; with (*N* = 257) and without type 2 diabetes (T2DM) (*N* = 329), (E) AST groups; < 40 (*N* = 324); ≥ 40 (*N* = 262), (F) ALT groups; < 45 (*N* = 199); ≥ 45 (*N* = 387). ELF, enhanced liver fibrosis; FIB‐4, Fibrosis‐4 Index; LSM, liver stiffness measurement.

The three tests performed differently when evaluated in the 586 included participants: ELF had an AUC of 0.79 (95% CI: 0.75–0.84), FIB‐4 an AUC of 0.74 (95% CI: 0.69–0.78), while VCTE LSM showed an AUC of 0.83 (95% CI: 0.79–0.86). The performance of the tests in different subgroups of patients is illustrated in Figure 2A–F. Overall, the diagnostic performance of the three tests in detecting F ≥ 3 was not significantly different between the respective subgroups (*p* > 0.05). For instance, in both males and females AUCs were around 0.80 for ELF, 0.73 for FIB‐4 and 0.83 for VCTE LSM. Despite slight differences in the AUC, the performance of all three tests was comparable in different age and BMI subgroups. No significant difference was observed in performance of the three tests in subgroups of patients with and without diabetes and in those with different levels of liver enzymes (Tables S4 and S5).

Adjusting for age, sex, BMI and T2DM in estimating covariate‐adjusted ROC curves did also not show statistically significant differences in AUC across subgroups (Table S4).

### 3.4 Desired Thresholds at Pre‐Defined Sensitivity and Specificity

Table 2 shows estimates of the tests' thresholds for fixed high sensitivity and specificity.

**TABLE 2:** Thresholds of the tests to detect advanced liver fibrosis in different subgroups to achieve fixed sensitivities and specificities.

			ELF thresholds for		FIB‐4 thresholds for		VCTE LSM thresholds for	
Subgroups	n	Prev%	Sensitivity 90%	Specificity 90%	Sensitivity 90%	Specificity 90%	Sensitivity 90%	Specificity 90%
Female	211	34	8.72	10.38	0.95	2.06	6.75	13.95
Male	375	25	8.59	9.95	0.83	2.07	7.65	14.05
Age 18–45	157	10	6.23	9.47	0.26	1.24	5.95	12.70
Age 45–65	331	33	8.59	10.06	0.90	1.97	7.85	14.05
Age > 65	98	42	9.49	10.66	1.20	3.04	6.95	14.40
BMI < 25	70	14	6.91	9.97	0.54	1.93	5.00	10.45
BMI 25–30	209	24	8.45	9.98	0.83	2.24	6.75	11.95
BMI ≥ 30	307	34	8.80	10.27	0.87	1.85	7.95	15.05
Non‐T2DM	329	14	7.31	9.98	0.41	1.95	6.75	11.95
T2DM	257	46	8.76	10.36	0.91	2.18	7.75	16.40
AST < 40	324	19	8.45	9.91	0.74	1.79	7.35	12.25
AST ≥ 40	262	39	8.79	10.36	1.06	2.50	7.65	15.25
ALT < 45	199	23	8.53	10.01	0.75	1.94	6.95	13.05
ALT ≥ 45	387	31	8.66	10.15	0.90	2.08	7.35	14.35

Abbreviations: ALT, alanine aminotransferase; AST, aspartate aminotransferase; BMI, body mass index; ELF, enhanced liver fibrosis; FIB‐4, Fibrosis‐4 Index; LSM, liver stiffness measurement; *n*, sample size; T2DM, type 2 diabetes.

#### 3.4.1 Sex at Birth (Female, Male)

Slightly higher thresholds of ELF and FIB‐4 were required to achieve a sensitivity of 90% in females (8.72 and 0.95 vs. 8.59 and 0.83, respectively). While for VCTE LSM, a higher threshold was observed in males (7.65 kPa vs. 6.75 kPa). To reach a 90% specificity the threshold of VCTE LSM had to be higher in males (VCTE LSM: 14.05 kPa vs. 13.95 kPa). The calculated ELF threshold was higher in females (10.38 vs. 9.95).

#### 3.4.2 Age Subgroups (18–45, 45–65, and > 65 Years)

We observed higher thresholds of ELF and FIB‐4 in higher age ranges while pre‐specifying the sensitivity level at 90%. Pre‐defining specificity at 90% resulted in higher thresholds of all three tests in higher age ranges.

#### 3.4.3 BMI Subgroups (< 25, 25–30 and BMI ≥ 30 kg/m^2^)

When pre‐specifying a 90% sensitivity, we observed higher thresholds for all three tests in patients with a higher BMI (BMI≥ 30) compared to those with a lower BMI (BMI 25–30 and < 25 kg/m^2^). For 90% specificity the same trend was observed for ELF and VCTE LSM.

#### 3.4.4 Diabetes Status (T2DM or No T2DM)

In patients with T2DM we observed higher thresholds for reaching 90% sensitivity (ELF: 8.76) and 90% specificity (VCTE LSM: 16.40 kPa) compared to non‐T2DM patients (ELF: 7.03, VCTE LSM: 12.95 kPa).

#### 3.4.5 Liver Enzymes, AST (< 40 or ≥ 40) and ALT (< 45 or ≥ 45)

Lower thresholds in the low AST group for 90% sensitivity (ELF: 8.45, VCTE LSM: 12.25 kPa) compared to the high AST group (ELF: 8.79, VCTE LSM: 15.25 kPa). Similar trend with ALT for 90% sensitivity and specificity.

### 3.5 Desired Thresholds to Rule In or Rule Out F ≥ 3

When we pre‐specified the PPV and NPV for ruling in or ruling out F ≥ 3, we found different threshold values for the tests (Table 3).

**TABLE 3:** Thresholds to detect advanced fibrosis in different subgroups to achieve pre‐defined predictive values.

		ELF thresholds for		FIB‐4 thresholds for		VCTE LSM thresholds for	
Subgroups	Prev%	PPV 70%	NPV 80%	PPV 70%	NPV 80%	PPV 70%	NPV 80%
Female	34	10.27	7.37	2.10	0.31	11.05	4.45
Male	25	10.15	6.33	3.77	0.32	18.70	4.45
Age 18–45	10	10.15	6.37	NA	0.32	17.65	5.95
Age 45–65	33	10.06	7.48	2.27	0.60	13.65	4.45
Age > 65	42	10.28	8.88	3.41	NA	12.25	4.90
BMI < 25	14	10.07	6.91	2.22	0.54	19.30	5.00
BMI 25–30	24	10.18	7.83	4.36	0.41	17.45	4.45
BMI ≥ 30	34	10.21	7.15	1.86	0.41	13.55	4.45
Non‐T2DM	14	12.03	6.90	3.28	0.33	19.40	4.45
T2DM	46	9.71	8.08	1.82	0.51	10.65	5.05
AST < 40	19	10.77	6.96	5.47	0.41	16.95	4.45
AST ≥ 40	39	10.17	8.03	2.35	0.54	13.20	4.45
ALT < 45	23	10.80	7.10	2.37	0.53	14.35	5.95
ALT ≥ 45	31	10.16	6.37	2.84	0.36	15.45	4.45

*Note:* NA: no threshold was found corresponding the fixed predictive value.

Abbreviations: ALT, alanine aminotransferase; AST, aspartate aminotransferase; BMI, body max index; ELF, enhanced liver fibrosis; FIB‐4, Fibrosis‐4 Index; LSM, liver stiffness measurement; PPV, positive predictive value; Prev, prevalence; NPV, negative predictive value; T2DM, type 2 diabetes.

#### 3.5.1 Sex at Birth (Female, Male)

To reach a PPV of 70% both FIB‐4 and VCTE LSM thresholds were higher in males, while the ELF threshold was higher for females (10. 278 vs. 10.15).

To reach a NPV of 80% the threshold of ELF was higher for females (7.37 vs. 6.33).

#### 3.5.2 Age Subgroups (18–45, 45–65 and > 65 Years)

In younger patients (18–45 years) a higher threshold of VCTE LSM was needed to reach the pre‐specified PPV of 70% (12.25 kPa vs. 17.65 kPa). In this group, lower thresholds of ELF resulted in an 80% NPV, compared to older patients, while for VCTE LSM the threshold for ruling out F ≥ 3 was highest in this subgroup.

#### 3.5.3 BMI Subgroups (< 25, 25–30 and BMI ≥ 30 kg/m^2^)

Lower thresholds of FIB‐4 and VCTE LSM were needed to achieve 70% PPV in higher BMI groups (VCTE LSM: 19.30 kPa < 25 BMI vs. 13.55 kPa ≥ 30 BMI) while the ELF threshold had to be higher. In obese and overweight patients lower FIB‐4 and VCTE LSM thresholds could result in 80% NPV.

#### 3.5.4 Diabetes Status (T2DM or No T2DM)

In patients with T2DM lower thresholds of all three tests were shown to be required for detecting F ≥ 3 with a PPV of 70%. In contrast, lower thresholds to reach NPV of 80% were observed for all tests in patients without T2DM.

#### 3.5.5 Liver Enzymes, AST (< 40 or ≥ 40) and ALT (< 45 or ≥ 45)

Thresholds for PPV and NPV varied based on AST and ALT levels, with in general, higher thresholds for reaching PPV of 70% in low AST (< 40 U/L) groups and lower thresholds for reaching a NPV of 80% in low AST and ALT (< 45 U/L) groups.

## 4 Discussion

Our stepwise analysis confirmed the practicability of the EASL‐recommended approach, where FIB‐4 effectively stratifies patients and imaging techniques, like VCTE, refine the assessment in the group with intermediate results. This aligns with EASL guidelines, which emphasise the value of such a strategy for identifying individuals with advanced fibrosis, who are at risk of developing liver‐related outcomes and guiding them into appropriate clinical care pathways. However, as our cohort primarily represents secondary and tertiary care settings, these findings may not be directly generalizable to the general population.

In addition, our analysis showed overall small differences in performance of the three NITs in detecting F ≥ 3 in different subgroups. However, the difference was more evident for some subgroups, such as ELF in the older versus younger patients.

Overall, the three NITs performed similarly in patients with and without T2DM. ELF and VCTE LSM demonstrated similar accuracy across BMI categories. However, patients with higher BMI and T2DM required higher thresholds for fixed sensitivity and specificity. Notably, VCTE LSM thresholds differed significantly between low (< 25 kg/m^2^) and high BMI (≥ 30 kg/m^2^) and between younger and older patients for fixed specificity.

This study is conducted as a proof‐of‐principle demonstration based on single features to provide a foundation for understanding the complex interaction between diagnostic test outcomes and diverse clinical characteristics. Building on initial findings from the LITMUS study [15], we analysed the data from the intended‐use population recruited from multiple secondary or tertiary care centres in Europe. In the recently published study from the LITMUS consortium, the performance of different NITs was assessed separately in individuals with and without diabetes [15]. The results showed marginal differences in the performance of tests among participants with and without diabetes, particularly in detecting MASH and F ≥ 2. In the current manuscript, we extend upon these preliminary findings by presenting a more comprehensive subgroup analysis, incorporating variables such as age, BMI and liver enzymes to emphasise the importance of a holistic understanding of diagnostic accuracy, acknowledging the variability in clinical factors. Nevertheless, we acknowledge some limitations. Sample size constraints, incomplete data and missing values from some centres highlight the challenges of conducting multicentre studies. In particular, the availability of test results was influenced by logistical constraints, such as sample availability for blood‐based markers and the capacity of local centres to perform FibroScan, limiting complete data collection across all patients in the cohort. Additionally, the absence of centralised histological scoring of liver biopsies may introduce potential variability in diagnostic precision [32]. Limitations of liver biopsy in general, an imperfect reference standard, may also have led to bias in the estimates of test accuracy [33, 34].

We also acknowledge the potential spectrum effect as the prevalence of F ≥ 3 was different in each subgroup evaluated in our analysis. However, the differences in the thresholds that were needed to achieve the pre‐specified level of sensitivity and specificity cannot be caused by differences in prevalence of the target condition across subgroups, as the accuracy statistics are based on conditional distributions: sensitivity is based on the distribution of test results in those with the target condition and specificity on the distribution in those without. These respective distributions differed somewhat across the respective subgroups.

While evaluating test performance we observed higher AUCs for all tests in older patients, especially patients between 45 and 65 years, in comparison to younger ones. One study, conducted in healthy volunteers, identified age as an important determinant of differences in ELF results, with higher reference values being observed in older age groups [20]. VCTE LSM is presumed to be an age‐independent test that can be used in older patients [35]. Our results underlined this age‐independency, as we observed more comparable findings for VCTE LSM across age categories.

There are also other published studies that suggest variations of NIT accuracy in patients with different basic clinical characteristics [36]. These were not always consistent in the conclusions drawn. One study suggested that NITs may be useful for identifying F ≥ 3 in patients with T2DM as well as patients without T2DM [37], while another study reported lower performance of NITs in patients with T2DM [38]. This may be due, in part, to other differences between subgroups under evaluation.

A recent study suggested that variations in NIT results between patients with and without diabetes may be influenced by differences in other patient characteristics. In that analysis ELF was not included and research into the performance of the ELF test in patients with T2DM was recommended [39]. In our study, we observed similar AUCs for ELF in patients with and without diabetes, as well as comparable performance across BMI categories. This might imply that ELF can be used as a suitable NIT in patients with MASLD, regardless of BMI and diabetes status. This paper, however, has a focus on sequential diagnostic algorithms in patients with and without T2DM but they do not look at different age, BMI and liver enzymes [39].

In contrast to sensitivity and specificity, predictive values are affected by differences in prevalence. We therefore also analysed what thresholds would be able to rule in or rule out the disease. Our findings showed varying thresholds for each test in different subgroups to achieve desired predictive values. These differences are based both on the difference in prevalence and on the shift in the distribution of test results in participants with or without the target condition (Figure S1). As expected, lower thresholds were needed to achieve the desired PPV in patients with higher age, with diabetes and with obesity, where the prevalence of F ≥ 3 was higher. In overweight patients, however, higher thresholds were required to result in the same level of PPV, despite the higher prevalence of F ≥ 3, compared to patients with normal BMI. These findings highlight that a single pre‐defined threshold of a test would not result in similar predictive values in patients that differ in basic clinical characteristics.

As age is included as one of the components of the FIB‐4 algorithm, we observed higher FIB‐4 scores in elderly patients, and consequently, higher thresholds were needed to rule out F ≥ 3. Some studies proposed adapting a different threshold of FIB‐4 in older patients that could reduce the need for unnecessary biopsies and inappropriate referrals [19]. However, these age‐specific thresholds should be used cautiously as evidence shows that they may result in a considerable concomitant decrease in sensitivity for the diagnosis of F ≥ 3 in older patients [40]. Therefore, current guidelines suggest using FIB‐4 as a first test with the same threshold in all patients and then confirming with an age‐independent test [35].

Guidelines suggest NITs to differentiate low and high‐risk patients for F ≥ 3 [5, 6, 28], but currently overlook variations in basic clinical or demographic characteristics. The thresholds identified in this study are reported to illustrate the implications of considering clinical and demographic characteristics. However, they were not independently validated, and each considers only a single variable at a time. Sample size limitations prevented us from performing multivariable analyses. New and larger studies, such as the one currently performed by the LITMUS consortium, are anticipated to provide the necessary data for identifying multivariable thresholds that consider multiple characteristics simultaneously and validate these in an independent dataset.

## 5 Conclusion

In conclusion, this study highlights the importance of understanding diagnostic accuracy across diverse patient groups with MASLD. While the performance of FIB‐4, ELF and VCTE LSM in detecting advanced liver fibrosis in our MASLD patients did not vary substantially by age, sex, BMI and presence of T2DM, different thresholds were needed to achieve the same sensitivity, specificity and predictive values across clinical subgroups. This highlights the fact that the accuracy of NITs can vary in specific demographic groups and can be optimised by using adjusted thresholds. Clinicians should keep this in mind when using NITs in clinical practice, where different positivity thresholds are required, depending on basic patient characteristics.

## Author Contributions

P.M.B., A.G.H., Y.V. and A.‐M.v.D. conceptualised and designed the study; A.‐M.v.D and Y.V. performed data analyses and drafted the manuscript; P.M.B. supervised the data analyses and A.G.H. commented on the data analyses; all authors (A.‐M.v.D, Y.V., J.L., J.B., V.R., C.Y., J.M.S., L.V., M.R.G., D.S., S.P., M.A., M.L.H., K.P., J.F.D., E.B., A.G., Z.D., C.F.P., E.S.H., M.K., H.Y., M.E., A.G., A.T., S.F., C.B., M.P., A.G.H., M.N., Q.M.A., P.M.B.) critically revised the manuscript, approved of the final version, including the authorship list, and had final responsibility for the decision to submit for publication.

## Ethics Statement

The contributing projects were approved by the relevant Ethical Committees in the participating countries, and studies were conducted according to the guidelines of the Declaration of Helsinki. Name of the ethical committees by country: Belgium: Ethisch Comité van het Universitair Ziekenhuis Antwerpen. Finland: HUS eettinen toimikunta IV. France: Comite de Protection des Personnes (CPP) PP Ille‐de‐France VI, Comite de Protection des Personnes (CPP) Sud Mediterranee IV, Comite de Protection des Personnes (CPP) Ouest II—Angers. Germany: Ethikkommission der Landesärztekammer Rheinland‐Pfalz, Ethikkommission Medizinische Hochschule Hannover, Ethik‐Kommission der Universität Würzburg. Greece: General Hospital of Athens ‘Laiko’. Italy: A.O.U. City of Health and Science of Turin—A.O. Mauritian Order—ASL TO 1, Comitato Ethico Palermo, Asienda OspedaieraUniversitaria Policlinicl Paolo Giaccone di Palermo, Comitato Etico Fondazione Policlinico Gemelli IRCCS. Roma. Netherlands: Medisch Ethische Toetsings Commissie AMC. Portugal: Comissão de Ética do Centro Hospitalar Lisboa Norte (CHLN) e do Centro Académico de Lisboa (CAML). Spain: CEI de los Hospitales Universitarios Virgen Macarena y Virgen del Rocío. Sweden: Regionala etikpövningsnämnden I Linköping. Switzerland: Kantonale Ethikkommission Bern (KEK). UK: NRES Committee North East—Tyne & Wear South.

## Consent

All participants, initially recruited during two EU‐funded projects, provided informed consent prior to inclusion.

## Conflicts of Interest

QMA is coordinator of the IMI2 LITMUS consortium funded by the Innovative Medicines Initiative (IMI2) Program of the European Union under Grant Agreement 777 377. This multi‐stakeholder consortium includes industry partners and received funding from EFPIA. He reports research grant funding from Abbvie, AstraZeneca, Boehringer Ingelheim, Glympse Bio, Intercept, Novartis, Pfizer; consultancy on behalf of Newcastle University for Alimentiv, Akero, AstraZeneca, Axcella, 89Bio, Boehringer Ingelheim, Bristol Myers Squibb, Galmed, Genfit, Genentech, Gilead, GlaxoSmithKline, Hanmi, HistoIndex, Intercept, Inventiva, Ionis, IQVIA, Janssen, Madrigal, Medpace, Merck, NGMBio, Novartis, Novo Nordisk, PathAI, Pfizer, Poxel, Resolution Therapeutics, Roche, Ridgeline Therapeutics, RTI, Shionogi, Terns; and speaker fees from Fishawack, Integritas Communications, Kenes, Novo Nordisk, Madrigal, Medscape, Springer Healthcare. Advisory board: On behalf of Newcastle University from Medpace (North Sea Therapeutics DSMB); licences: Elsevier Ltd. JB reports research grant funding (unrelated to this project) from EchoSens, Intercept, Inventiva, Siemens; consulting fees from Echosens, Intercept, Siemens; speaker fees from Gilead, Intercept, Lilly, Siemens; participation on the advisory board of Bristol‐Myers, Echosens, Intercept, Novo Nordisk, MSD. AG reports support from the IMI2 LITMUS project; research grants from Novartis, Falk and Intercept; consulting or speakers fees from Abbvie, Advanz, Albireo, Alexion, AstraZeneca, Bayer, BMS, Boehringer, Burgerstein, CSL Behring, Eisai, Falk, Gilead, Heel, Intercept, Ipsen, Merz, MSD, Novartis, NovoNordisk, Orphalan, Pfizer, Roche, Sanofi‐Aventis; and travel/meeting supports from Intercept, Gilead, Abbvie and Falk. SF holds a senior clinical investigator fellowship from the Research Foundation Flanders (FWO) (1 802 154 N). His institution has received grants from Genfit, Gilead Sciences, Roche and Bristol‐Meyer Squibb. SF has acted as consultant for Roche Intercept, Gilead Sciences Echosens, Allergan Genentech, Abbvie Novo Nordisk, Bayer Novartis, Bristol‐Meyers Squibb Astra Zeneca, Boehringer Ingelheim Galmed, Merck Sharp & Dohme, Promethera, Janssen Pharmaceutica Coherus, Actelion Madrigal, Astellas Julius Clinical, Genfit, NGM Bio, Inventiva. SF has been lecturer for Abbvie, Allergan, Bayer, Genfit, Gilead Sciences. MA reports research grants from GSK Takeda and Astra Zeneca, and consulting fees from Intercept and Astra Zeneca. MN is co‐founder and member of the Scientific Advisory Board of Caelus Pharmaceuticals and Advanced Microbiota Therapeutics, the Netherlands. None of these are directly relevant to the current paper. JFD participates on the advisory committees of Alentis, Astra‐Zeneca, Bayer, Bristol‐Myers Squibb, Enyo, Esai, Falk, Genfit, Gilead Sciences, Intercept, Inventiva, Ipsen, Lilly, Madrigal, Merck, Novartis, Novo‐Nordisk, Roche. She reports travel/ meeting support from Gilead; speaking and teaching fees from Bayer, Bristol‐Myers Squibb, Intercept, Gilead Sciences, Novartis, Roche. Licences: GUT as AE, Uptodate. She is president of the Swiss NASH Foundation and the Swiss Foundation against Liver Cancer. MP reports support from the IMI2 LITMUS project; grants from Cancer Research UK. Patents: Medical Imaging, International publication, number WO2015155521A1. He is a shareholder in Perspectum Ltd. HYJ reports support from the IMI2 LITMUS project; grants from Academy of Finland, Sigrid Juselius Foundation and EVO foundation; consulting fee from Boehringer Ingelheim; speaker fee from Novo Nordisk and Glaxo; Advisory board of MSD, Eli Lilly and Hamni. CY is an employee and stock shareholder of Pfizer Inc. CB is former employee of Novartis Pharma. He reports financial support from Novartis Pharma. Patents: Pending patents for treatment of NASH (Novartis). Stock: Novartis Pharma, Merck. ME participates on the advisory board of AMRA Medical. JS reports grants from Gilead Sciences, Boehringer Ingelheim, Nordic Bioscience, Siemens Healthcare GmbH; consulting fees from Apollo Endosurgery, Albireo Pharma Inc., Bayer, BMS, Boehringer Ingelheim, Echosens, Genfit, Gilead Sciences, GSK, Heel GmbH, Intercept Pharmaceuticals, Ipsen, Inventiva Pharma, Julius Clinical, Madrigal, MSD, Nordic Bioscience, Novartis, Novo Nordisk, Pfizer, Roche, Sanofi, Shinogi, Siemens Healthcare GmbH, Summit Clinical Research; speaker fee from Apollo Endosurgery, MedPublico GmbH, Boehringer Ingelheim. He is on the advisory board of Novo Nordisk, Boehringer Ingelheim. EB reports consulting fees from Gilead, Novo Nordisk, Boehringer, Intercept; speaker fees from Novo Nordisk, Intercept, MDS. She is on the advisory board of Novo Nordisk, Intercept. VR reports grants from Gilead Sciences, Intercept Pharmaceuticals; consulting fees from Glaxo‐Smith‐Kline, Galmed, Novo‐Nordisk, Prosciento, Terns, NorthSea Therapeutics, Enyo, Sagimet, NGM Pharmaceuticals, Madrigal. MRG reports grants from Siemens, Gilead, Intercept; consulting fee from Abbvie, Alpha‐sigma, Allergen, Astra‐Zeneca, Inventia, Kaleido, Novo Nordisk, Pfizer, Axcella, BMS, Boehringer‐ingelheim, Gilead; speaker fee from Inventia, Sobi, Novo Nordisk, Rubio, Shionogi; meeting/travel support from Abbvie and Gilead; advisory board of Galmed. Other authors do not have competing interest.

## Supporting information


Data S1.

## Data Availability

At present, data from this study are not publicly available. The LITMUS study protocol has been published, including details of sample handling and processing, and includes an atlas of liver histology that may be used as a reference by pathologists. Patient level data will not be made available due to the various constraints imposed by ethics panels across all the different countries from which patients were recruited.
