# Supplementary material for: Precision in Liver Diagnosis: Varied Accuracy Across Subgroups and the Need for Variable Thresholds in Diagnosis of MASLD
Source: Liver Int. 2025 Jan 24;45(2):e16240. doi: 10.1111/liv.16240 (PMC11771619; doi:10.1111/liv.16240)
Supplement: Supplementary file 1 — Data S1. [file LIV-45-0-s001.docx]

# Supplementary material

**Supplementary table 1:** The LITMUS investigators

| **The LITMUS Investigators / Group Authors** | |
| --- | --- |
| Newcastle University | Quentin M. Anstee  Ann K. Daly  Olivier Govaere  Simon Cockell  Dina Tiniakos †  Pierre Bedossa †  Alastair Burt †  Fiona Oakley  Heather J. Cordell  Christopher P. Day  Kristy Wonders  Paolo Missier  Matthew McTeer  Luke Vale  Yemi Oluboyede  Matt Breckons |
| AMC Amsterdam | Patrick M. Bossuyt  Hadi Zafarmand  Yasaman Vali  Jenny Lee  Max Nieuwdorp  Adriaan G. Holleboom  Joanne Verheij † |
| Institute of Cardiometabolism And Nutrition | Vlad Ratziu  Karine Clément  Rafael Patino-Navarrete  Raluca Pais |
| Hôpital Beaujon, Assistance Publique Hopitaux de Paris | Valerie Paradis † |
| University Medical Center Mainz | Detlef Schuppan  Jörn M. Schattenberg  Rambabu Surabattula  Sudha Myneni  Beate K. Straub † |
| University of Cambridge | Toni Vidal-Puig  Michele Vacca  Sergio Rodrigues-Cuenca  Mike Allison  Ioannis Kamzolas  Evangelia Petsalaki  Mark Campbell  Chris J. Lelliott  Susan Davies † |
| Örebro University | Matej Orešič  Tuulia Hyötyläinen  Aiden McGlinchey |
| Center for Cooperative Research in Biosciences | Jose M. Mato  Óscar Millet |
| University of Bern | Jean-François Dufour  Annalisa Berzigotti  Mojgan Masoodi |
| University of Oxford | Michael Pavlides  Stephen Harrison  Stefan Neubauer  Jeremy Cobbold  Ferenc Mozes  Salma Akhtar  Seliat Olodo-Atitebi |
| Perspectum | Rajarshi Banerjee  Matt Kelly  Elizabeth Shumbayawonda  Andrea Dennis  Anneli Andersson  Ioan Wigley |
| Servicio Andaluz de Salud, Seville | Manuel Romero-Gómez  Emilio Gómez-González  Javier Ampuero  Javier Castell  Rocío Gallego-Durán  Isabel Fernández  Rocío Montero-Vallejo |
| Nordic Bioscience | Morten Karsdal  Daniel Guldager Kring Rasmussen  Diana Julie Leeming  Antonia Sinisi  Kishwar Musa |
| Integrated Biobank of Luxembourg | Estelle Sandt  Manuela Tonini |
| University of Torino | Elisabetta Bugianesi  Chiara Rosso  Angelo Armandi |
| Università degli Studi di Firenze | Fabio Marra |
| Consiglio Nazionale delle Ricerche | Amalia Gastaldelli |
| Università Politecnica delle Marche | Gianluca Svegliati |
| University Hospital of Angers | Jérôme Boursier |
| Antwerp University Hospital | Sven Francque  Luisa Vonghia  Ann Driessen † |
| Linköping University | Mattias Ekstedt  Stergios Kechagias |
| University of Helsinki | Hannele Yki-Järvinen  Kimmo Porthan  Johanna Arola † |
| UMC Utrecht | Saskia van Mil |
| Medical School of National & Kapodistrian University of Athens | George Papatheodoridis |
| Faculdade de Medicina, Universidade de Lisboa | Helena Cortez-Pinto |
| Faculty of Pharmacy, Universidade de Lisboa | Cecilia M. P. Rodrigues |
| Università degli Studi di Milano | Luca Valenti  Serena Pelusi |
| Università degli Studi di Palermo | Salvatore Petta  Grazia Pennisi |
| Università Cattolica del Sacro Cuore | Luca Miele |
| University Hospital Würzburg | Andreas Geier |
| RWTH Aachen University Hospital | Christian Trautwein  Johanna Reißing |
| University of Nottingham | Guruprasad P. Aithal  Susan Francis  Naaventhan Palaniyappan  Christopher Bradley |
| Antaros Medical | Paul Hockings  Moritz Schneider |
| University Hospitals Birmingham NHS Foundation Trust | Philip Newsome  Stefan Hübscher † |
| iXscient | David Wenn |
| Genfit | Christian Rosenquist |
| Intercept Pharma | Aldo Trylesinski |
| OWL | Rebeca Mayo  Cristina Alonso |
| Eli Lilly and Company | Kevin Duffin  James W. Perfield  Yu Chen |
| Pfizer | Carla Yunis  Theresa Tuthill  Magdalena Alicia Harrington  Melissa Miller  Yan Chen  Euan James McLeod  Trenton Ross  Barbara Bernardo |
| Boehringer-Ingelheim | Corinna Schölch  Judith Ertle  Ramy Younes  Anouk Oldenburger  Harvey Coxson |
| Somalogic | Rachel Ostroff  Leigh Alexander  Hannah Biegel |
| Novo Nordisk | Mette Skalshøi Kjær  Lea Mørch Harder  Peter Davidsen |
| Ellegaard Göttingen Minipigs | Jens Ellegaard |
| Novartis Pharma AG | Maria-Magdalena Balp  Clifford Brass  Lori Jennings  Miljen Martic  Jürgen Löffler  Douglas Applegate |
| AstraZeneca | Sudha Shankar  Richard Torstenson  Daniel Lindén |
| Echosens | Céline Fournier-Poizat  Anne Llorca |
| Resoundant | Michael Kalutkiewicz  Kay Pepin  Richard Ehman |
| Bristol-Myers Squibb | Gerald Horan |
| HistoIndex | Gideon Ho  Dean Tai  Elaine Chng |
| Gilead | Scott D. Patterson  Andrew Billin |
| RTI-HS | Lynda Doward  James Twiss |
| Takeda Pharmaceuticals Company Ltd. | Paresh Thakker  Zoltan Derdak |
| AbbVie | Henrik Landgren |
| Medical University of Graz | Carolin Lackner † |
| University of Groningen | Annette Gouw † |
| Aristotle University of Thessaloniki | Prodromos Hytiroglou † |

† Member of the LITMUS Histopathology Group (LHG)

**Supplementary Table 2**. Characteristics of the study group of patients with NAFLD.

|  | Entire metacohort  (n=966) | Group with ELF^TM^ data  (n=906) | Group with FIB-4 data  (n=953) | Group with VCTE LSM data  (n=626) |
| --- | --- | --- | --- | --- |
| Age. years | 51.2 (13.0) | 51.1 (13.1) | 51.2 (13.0) | 52.0 (13.2) |
| Female | 403 (42%) | 377 (42%) | 395 (41%) | 230 (37%) |
| BMI. kg/m² | 34.12 (8.26) | 33.9 (7.83) | 33.9 (7.78) | 31.3 (6.01) |
| Diabetes | 406 (42%) | 378 (42%) | 405 (43%) | 284 (45%) |
| ALT. U/L | 62.7 (42.5) | 62.4 (40.8) | 62.6 (41.0) | 66.0 (40.1) |
| AST. U/L | 42.9 (26.0) | 42.6 (25.5) | 42.9 (25.6) | 44.4 (25.5) |
| GGT. U/L | 108 (155) | 105 (152) | 108 (155) | 102 (114) |
| Albumin. g/L | 4.38 (0.419) | 4.38 (0.417) | 4.39 (0.413) | 4.38 (0.402) |
| Platelet count. 10^9/L | 239 (73.4) | 239 (73.2) | 238 (73.0) | 235 (69.6) |
| Glucose. mmol/L | 6.42 (2.47) | 6.39 (2.40) | 6.44 (2.39) | 6.40 (2.37) |
| Triglycerides. mg/L | 2.08 (1.18) | 2.01 (1.01) | 2.09 (1.19) | 2.14 (1.28) |
| Liver fibrosis stage |  |  |  |  |
| 0 | 309 (32%) | 296 (33%) | 302 (32%) | 157 (25%) |
| 1 | 186 (19%) | 173 (19%) | 184 (19%) | 130 (21%) |
| 2 | 198 (21%) | 191 (21%) | 197 (21%) | 152 (24%) |
| 3 | 188 (19%) | 169 (19%) | 186 (20%) | 139 (22%) |
| 4 | 85 (9%) | 77 (8%) | 84 (9%) | 48 (8%) |
| NASH* | 512 (53%) | 473 (52%) | 509 (53%) | 351 (56%) |

All continuous variables are expressed as mean (SD). ELF^TM^. Enhanced Liver Fibrosis; FIB-4. Fibrosis-4 Index; VCTE LSM. Vibration Controlled Transient Elastography Liver Stiffness Measurement; NASH. non-alcoholic Steatohepatitis

* NAS score of ≥4 with at least one point in each component (inflammation. ballooning. steatosis)

**Supplementary Table 3**. Characteristics of patients in each subgroup (from total 586 patients)

|  | Sex | | |  | Age | | |  | BMI | | |  | Diabetes | | |  | AST | | |  | ALT | | | |
| --- | --- | --- | --- | --- | --- | --- | --- | --- | --- | --- | --- | --- | --- | --- | --- | --- | --- | --- | --- | --- | --- | --- | --- | --- |
|  | Female  (N=211) | Male  (N=375) | P-value |  | Age 18-65  (N=488) | Age >65  (N=98) | P-value |  | BMI <25  (N=70) | BMI ≥25  (N=516) | P-value |  | T2DM –  (N=329) | T2DM+  (N=257) | P-value |  | AST <40  (N=324) | AST ≥40  (N=262) | P-value |  | ALT <45  (N=199) | ALT ≥45  (N=387) | P-value |  |
| Age (Years) |  |  |  |  |  |  |  |  |  |  |  |  |  |  |  |  |  |  |  |  |  |  |  |  |
| Mean (SD) | 56.1 (11.9) | 49.7 (13.3) | <0.001 |  | - | - |  |  | 49.0 (14.7) | 52.4 (12.9) | 0.0686 |  | 47.6 (13.3) | 57.5 (10.7) | <0.001 |  | 52.2 (12.8) | 51.7 (13.7) | 0.678 |  | 55.7 (11.6) | 50.0 (13.5) | <0.001 |  |
| Sex |  |  |  |  |  |  |  |  |  |  |  |  |  |  |  |  |  |  |  |  |  |  |  |  |
| Female | - | - |  |  | 162 (33.2%) | 49.0 (50.0%) | 0.00231 |  | 17.0 (24.3%) | 194 (37.6%) | 0.0409 |  | 99.0 (30.1%) | 112 (43.6%) | 0.00101 |  | 112 (34.6%) | 99.0 (37.8%) | 0.471 |  | 93.0 (46.7%) | 118 (30.5%) | <0.001 |  |
| Male | - | - |  |  | 326 (66.8%) | 49.0 (50.0%) |  |  | 53.0 (75.7%) | 322 (62.4%) |  |  | 230 (69.9%) | 145 (56.4%) |  |  | 212 (65.4%) | 163 (62.2%) |  |  | 106 (53.3%) | 269 (69.5%) |  |  |
| BMI |  |  |  |  |  |  |  |  |  |  |  |  |  |  |  |  |  |  |  |  |  |  |  |  |
| Mean (SD) | 32.5 (6.60) | 30.6 (5.56) | <0.001 |  | 31.4 (6.20) | 30.6 (5.01) | 0.165 |  | - | - |  |  | 30.2 (5.86) | 32.6 (5.99) | <0.001 |  | 31.2 (6.32) | 31.4 (5.64) | 0.64 |  | 31.6 (6.65) | 31.1 (5.67) | 0.4 |  |
| T2DM |  |  |  |  |  |  |  |  |  |  |  |  |  |  |  |  |  |  |  |  |  |  |  |  |
| No | 99.0 (46.9%) | 230 (61.3%) | 0.00101 |  | 293 (60.0%) | 36.0 (36.7%) | <0.001 |  | 51.0 (72.9%) | 278 (53.9%) | 0.00404 |  | - | - |  |  | 186 (57.4%) | 143 (54.6%) | 0.547 |  | 98.0 (49.2%) | 231 (59.7%) | 0.0201 |  |
| Yes | 112 (53.1%) | 145 (38.7%) |  |  | 195 (40.0%) | 62.0 (63.3%) |  |  | 19.0 (27.1%) | 238 (46.1%) |  |  | - | - |  |  | 138 (42.6%) | 119 (45.4%) |  |  | 101 (50.8%) | 156 (40.3%) |  |  |
| ALT (U/L) |  |  |  |  |  |  |  |  |  |  |  |  |  |  |  |  |  |  |  |  |  |  |  |  |
| Mean (SD) | 59.3 (36.1) | 70.3 (42.2) | 0.001 |  | 69.1 (41.4) | 52.4 (31.9) | <0.001 |  | 62.1 (45.8) | 66.9 (39.6) | 0.402 |  | 69.8 (42.8) | 61.9 (36.7) | 0.0169 |  | 45.1 (18.9) | 92.6 (44.3) | <0.001 |  | - | - |  |  |
| AST (U/L) |  |  |  |  |  |  |  |  |  |  |  |  |  |  |  |  |  |  |  |  |  |  |  |  |
| Mean (SD) | 46.6 (30.7) | 43.2 (22.4) | 0.155 |  | 44.8 (26.4) | 42.4 (22.0) | 0.334 |  | 38.4 (19.6) | 45.2 (26.4) | 0.00976 |  | 42.3 (20.7) | 47.2 (30.8) | 0.0289 |  | - | - |  |  | 29.0 (11.7) | 52.3 (27.4) | <0.001 |  |
| GGT (U/L) |  |  |  |  |  |  |  |  |  |  |  |  |  |  |  |  |  |  |  |  |  |  |  |  |
| Mean (SD) | 112 (133) | 92.6 (95.9) | 0.0661 |  | 98.4 (103) | 105 (146) | 0.674 |  | 96.9 (107) | 99.9 (111) | 0.828 |  | 92.8 (97.8) | 108 (125) | 0.109 |  | 75.2 (73.4) | 130 (139) | <0.001 |  | 71.9 (91.4) | 114 (117) | <0.001 |  |
| Albumin (g/L) |  |  |  |  |  |  |  |  |  |  |  |  |  |  |  |  |  |  |  |  |  |  |  |  |
| Mean (SD) | 4.27 (0.413) | 4.44 (0.392) | <0.001 |  | 4.42 (0.392) | 4.19 (0.436) | <0.001 |  | 4.48 (0.414) | 4.36 (0.406) | 0.0379 |  | 4.43 (0.412) | 4.31 (0.393) | <0.001 |  | 4.39 (0.373) | 4.36 (0.447) | 0.42 |  | 4.33 (0.380) | 4.41 (0.419) | 0.0215 |  |
| Platelet (10^9/L) |  |  |  |  |  |  |  |  |  |  |  |  |  |  |  |  |  |  |  |  |  |  |  |  |
| Mean (SD) | 254 (69.8) | 225 (68.7) | <0.001 |  | 240 (70.8) | 213 (64.1) | <0.001 |  | 230 (63.3) | 236 (71.4) | 0.447 |  | 236 (71.8) | 235 (68.7) | 0.762 |  | 238 (63.7) | 233 (78.1) | 0.437 |  | 243 (70.2) | 232 (70.4) | 0.0851 |  |
| Glucose (mmol/L) |  |  |  |  |  |  |  |  |  |  |  |  |  |  |  |  |  |  |  |  |  |  |  |  |
| Mean (SD) | 6.59 (2.25) | 6.19 (2.24) | 0.0485 |  | 6.26 (2.31) | 6.70 (1.90) | 0.059 |  | 5.66 (1.50) | 6.43 (2.32) | <0.001 |  | 5.20 (0.765) | 7.79 (2.65) | <0.001 |  | 6.09 (1.96) | 6.64 (2.55) | 0.00562 |  | 6.35 (2.18) | 6.33 (2.29) | 0.912 |  |
| TG (mg/L) |  |  |  |  |  |  |  |  |  |  |  |  |  |  |  |  |  |  |  |  |  |  |  |  |
| Mean (SD) | 2.08 (1.08) | 2.04 (1.10) | 0.721 |  | 2.07 (1.13) | 1.94 (0.881) | 0.26 |  | 1.79 (0.590) | 2.08 (1.13) | 0.00652 |  | 1.86 (0.836) | 2.27 (1.29) | <0.001 |  | 1.89 (0.881) | 2.27 (1.28) | <0.001 |  | 1.97 (1.13) | 2.09 (1.07) | 0.241 |  |
| Fibrosis |  |  |  |  |  |  |  |  |  |  |  |  |  |  |  |  |  |  |  |  |  |  |  |  |
| Advanced fibrosis | 72.0 (34.1%) | 93.0 (24.8%) | 0.0207 |  | 124 (25.4%) | 41.0 (41.8%) | 0.00149 |  | 10.0 (14.3%) | 155 (30.0%) | 0.0091 |  | 47.0 (14.3%) | 118 (45.9%) | <0.001 |  | 62.0 (19.1%) | 103 (39.3%) | <0.001 |  | 46.0 (23.1%) | 119 (30.7%) | 0.0645 |  |
| ELF |  |  |  |  |  |  |  |  |  |  |  |  |  |  |  |  |  |  |  |  |  |  |  |  |
| Mean (SD) | 9.49 (1.17) | 9.07 (1.10) | <0.001 |  | 9.06 (1.11) | 10.0 (0.963) | <0.001 |  | 8.69 (1.15) | 9.29 (1.12) | <0.001 |  | 8.86 (1.05) | 9.68 (1.09) | <0.001 |  | 8.92 (1.01) | 9.59 (1.19) | <0.001 |  | 9.10 (1.06) | 9.28 (1.18) | 0.0724 |  |
| FIB-4 |  |  |  |  |  |  |  |  |  |  |  |  |  |  |  |  |  |  |  |  |  |  |  |  |
| Mean (SD) | 1.52 (1.10) | 1.32 (0.886) | 0.0284 |  | 1.24 (0.838) | 2.18 (1.19) | <0.001 |  | 1.27 (0.972) | 1.41 (0.970) | 0.247 |  | 1.19 (0.799) | 1.65 (1.10) | <0.001 |  | 1.10 (0.631) | 1.75 (1.18) | <0.001 |  | 1.37 (0.934) | 1.41 (0.990) | 0.62 |  |
| VCTE LSM |  |  |  |  |  |  |  |  |  |  |  |  |  |  |  |  |  |  |  |  |  |  |  |  |
| Mean (SD) | 11.2 (7.92) | 10.5 (8.82) | 0.337 |  | 10.3 (7.85) | 12.9 (11.0) | 0.0289 |  | 7.99 (6.97) | 11.1 (8.63) | <0.001 |  | 8.22 (4.16) | 14.0 (11.2) | <0.001 |  | 9.30 (6.79) | 12.5 (9.96) | <0.001 |  | 10.3 (8.97) | 11.0 (8.26) | 0.36 |  |

All continuous variables are expressed as mean (SD). ELF. Enhanced Liver Fibrosis; FIB-4. Fibrosis-4 Index; LSM. Liver Stiffness Measurement; T2DM. Type 2 Diabetes; TG, Triglyceride

**Supplementary table 4:** Unadjusted and covariate-adjusted AUC and 95% CI in all subgroups for all three NITs for detecting F≥3.

|  | **ELF**^TM^ | | |  | **FIB-4** | |  | **VCTE LSM** | |
| --- | --- | --- | --- | --- | --- | --- | --- | --- | --- |
|  | **N** | **AUC**  **(95% CI)** | **AAUC**  **(95% CI)** |  | **AUC**  **(95% CI)** | **AAUC**  **(95% CI)** |  | **AUC**  **(95% CI)** | **AAUC**  **(95% CI)** |
| **Female** | 211 | 0.77 (0.70-0.84) | 0.69 (0.60-0.78) |  | 0.73 (0.65-0.80) | 0.65 (0.56-0.74) |  | 0.83 (0.77-0.89) | 0.81 (0.73-0.87) |
| **Male** | 375 | 0.80 (0.74-0.85) | 0.75 (0.69-0.81) |  | 0.74 (0.68-0.80) | 0.70 (0.63-0.76) |  | 0.82 (0.78-0.87) | 0.80 (0.74-0.85) |
| **Age 18-45** | 157 | 0.70 (0.50-0.89) | 0.70 (0.45-0.89) |  | 0.67 (0.50-0.84) | 0.78 (0.56-0.92) |  | 0.77 (0.66-0.87) | 0.79 (0.61-0.92) |
| **Age 45-65** | 331 | 0.76 (0.70-0.82) | 0.71 (0.61-0.80) |  | 0.70 (0.64-0.76) | 0.64 (0.53-0.73) |  | 0.84 (0.79-0.88) | 0.80 (0.72-0.86) |
| **Age >65** | 98 | 0.77 (0.68-0.87) | 0.73 (0.66-0.79) |  | 0.64 (0.53-0.75) | 0.70 (0.62-0.76) |  | 0.76 (0.66-0.86) | 0.79 (0.73-0.85) |
| **BMI<25** | 70 | 0.78 (0.59-0.97) | 0.66 (0.47-0.82) |  | 0.78 (0.60-0.96) | 0.63 (0.46-0.78) |  | 0.79 (0.63-0.95) | 0.75 (0.64-0.85) |
| **BMI 25-30** | 209 | 0.78 (0.69-0.86) | 0.73 (0.67-0.79) |  | 0.72 (0.64-0.80) | 0.70 (0.64-0.76) |  | 0.82 (0.76-0.89) | 0.82 (0.77-0.87) |
| **BMI≥30** | 307 | 0.79 (0.73-0.84) | 0.74 (0.62-0.83) |  | 0.75 (0.69-0.80) | 0.65 (0.52-0.75) |  | 0.82 (0.78-0.87) | 0.74 (0.63-0.84) |
| **T2DM** | 257 | 0.77 (0.71-0.83) | 0.71 (0.64-0.78) |  | 0.71 (0.64-0.77) | 0.65 (0.57-0.73) |  | 0.79 (0.74-0.85) | 0.79 (0.73-0.84) |
| **non-T2DM** | 329 | 0.74 (0.65-0.83) | 0.74 (0.66-0.81) |  | 0.70 (0.62-0.79) | 0.71 (0.63-0.79) |  | 0.80 (0.73-0.87) | 0.81 (0.74-0.87) |
| **AST <40** | 324 | 0.78 (0.71-0.85) | 0.70 (0.62-0.78) |  | 0.68 (0.61-0.75) | 0.57 (0.48-0.66) |  | 0.83 (0.77-0.89) | 0.83 (0.77-0.89) |
| **AST ≥40** | 262 | 0.77 (0.71-0.83) | 0.68 (0.61-0.76) |  | 0.73 (0.67-0.80) | 0.66 (0.58-0.73) |  | 0.81 (0.75-0.86) | 0.81 (0.75-0.86) |
| **ALT <45** | 199 | 0.79 (0.71-0.86) | 0.74 (0.65-0.82) |  | 0.69 (0.60-0.78) | 0.69 (0.59-0.78) |  | 0.84 (0.78-0.90) | 0.84 (0.78-0.90) |
| **ALT ≥45** | 387 | 0.80 (0.74-0.85) | 0.71 (0.64-0.77) |  | 0.76 (0.71-0.81) | 0.67 (0.60-0.73) |  | 0.82 (0.77-0.86) | 0.82 (0.77-0.86) |

AUC. Area Under the Curve; AAUC. Adjusted AUC; CI. Confidence Interval; ELF^TM^. Enhanced Liver Fibrosis; FIB-4. Fibrosis-4 Index; VCTE LSM. Vibration Controlled Transient Elastography Liver Stiffness Measurement; BMI. Body Mass Index; T2DM. Type 2 Diabetes

**Supplementary table 5:** Comparison of non-invasive tests’ performance (AUCs) between different subgroups of patients.

| **Comparisons** | **P-Values** | | |
| --- | --- | --- | --- |
|  | **ELF** | **FIB-4** | **VCTE LSM** |
| Male vs Female | 0.58 | 0.75 | 0.85 |
| Age 18-45 vs Age 45-65 | 0.52 | 0.75 | 0.24 |
| Age 18-45 vs Age >65 | 0.47 | 0.78 | 0.93 |
| Age 45-65 vs Age >65 | 0.82 | 0.36 | 0.17 |
| BMI <25 vs BMI 25-30 | 0.96 | 0.57 | 0.71 |
| BMI <25 vs BMI >30 | 0.98 | 0.75 | 0.69 |
| BMI 25-30 vs BMI >30 | 0.87 | 0.60 | 0.97 |
| T2DM vs Non-T2DM | 0.55 | 0.98 | 0.89 |
| AST >40 vs AST <40 | 0.88 | 0.28 | 0.56 |
| ALT >45 vs ALT <45 | 0.84 | 0.22 | 0.57 |

**Supplementary figure 1:** Distribution of ELF, FIB-4 and VCTE LSM values by histological fibrosis stage in different **a)** sex, **b)** age, **c)** BMI, **d)** T2DM, **e)** AST and f**)** ALT subgroups

**1a)**


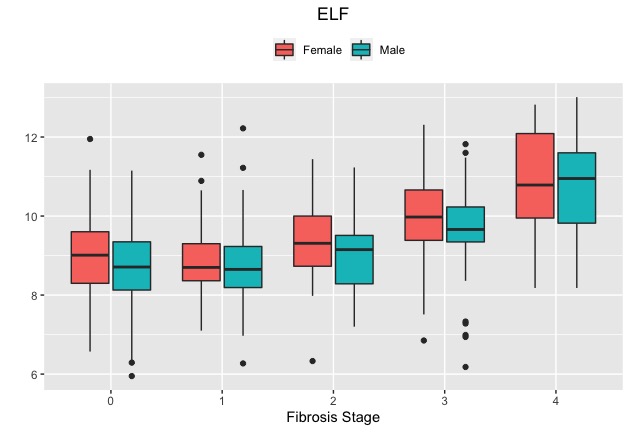


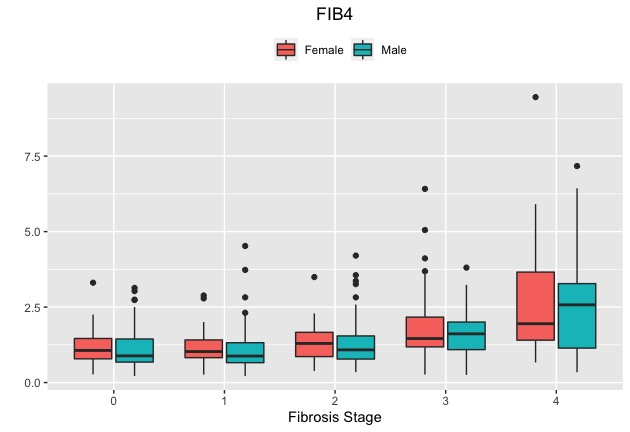

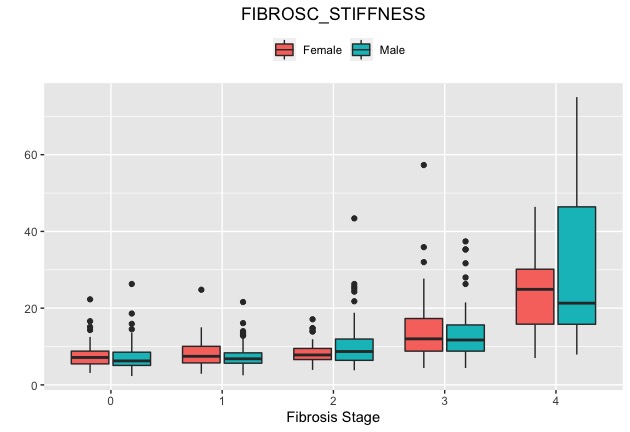


**1b)**

**
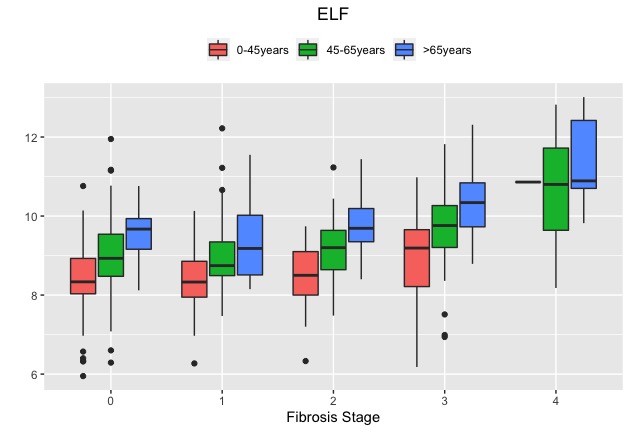
**

**
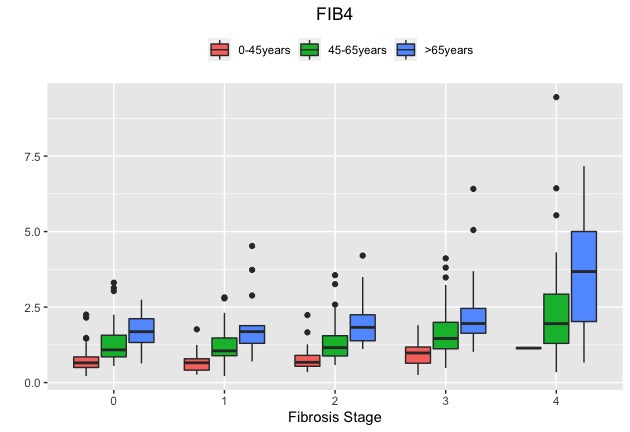
**

**
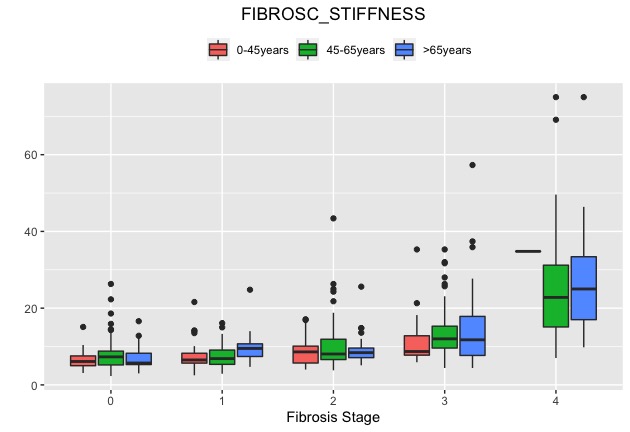
**

**1c)**


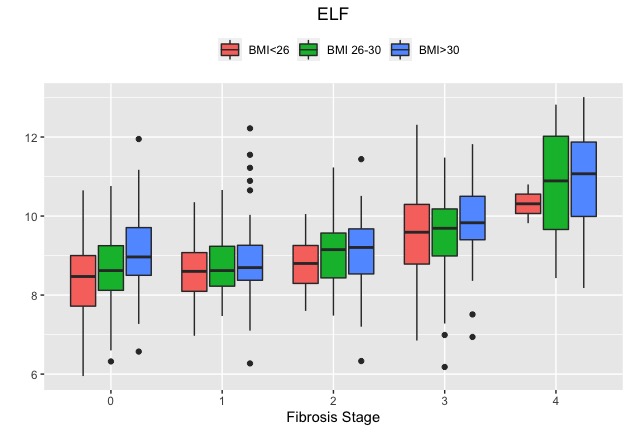


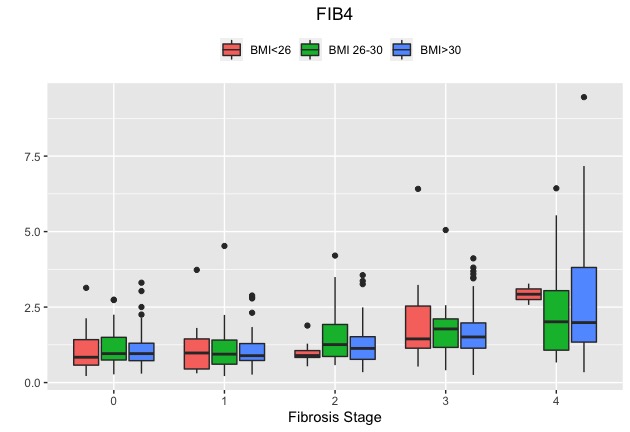


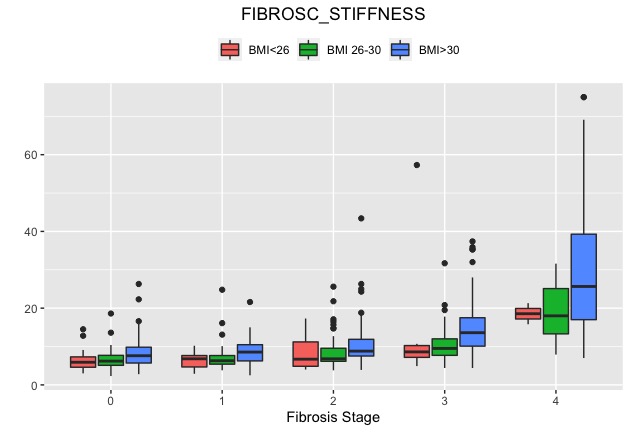


**1d)**

**
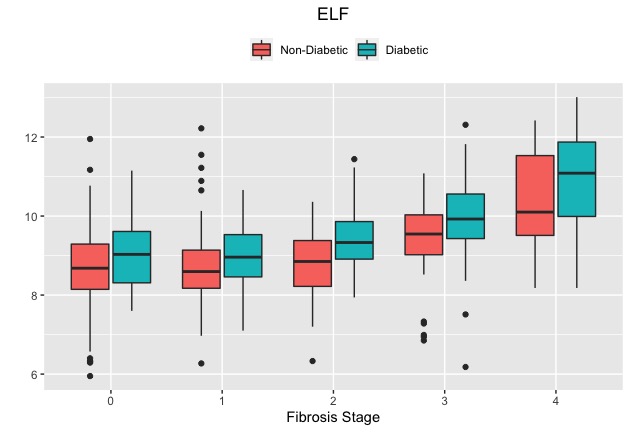
**


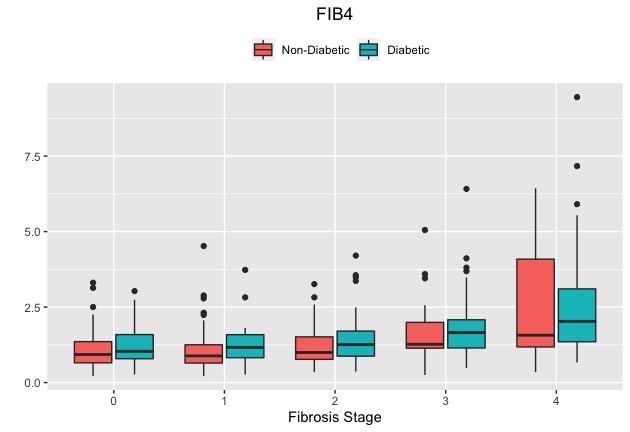


**
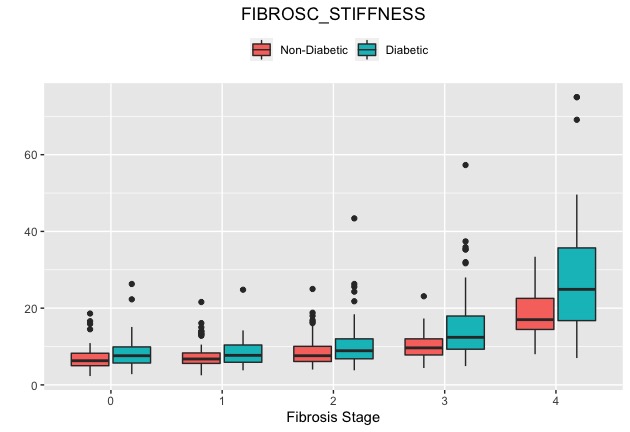
**

**1e)**

**
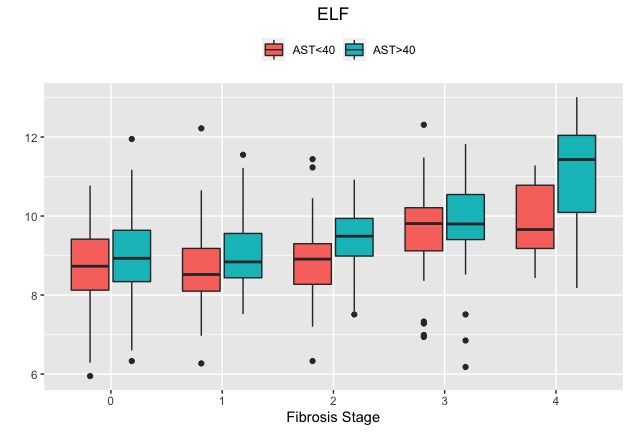
**


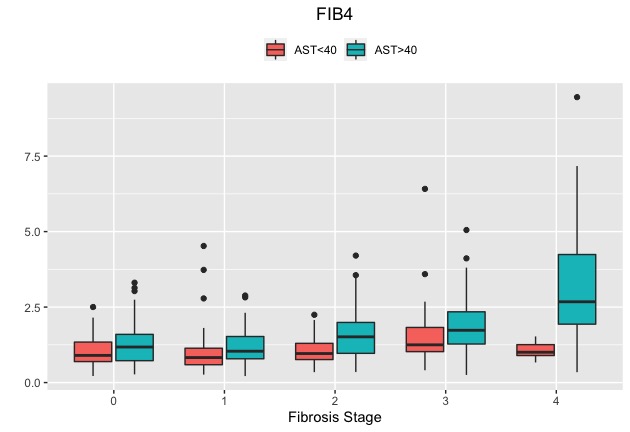


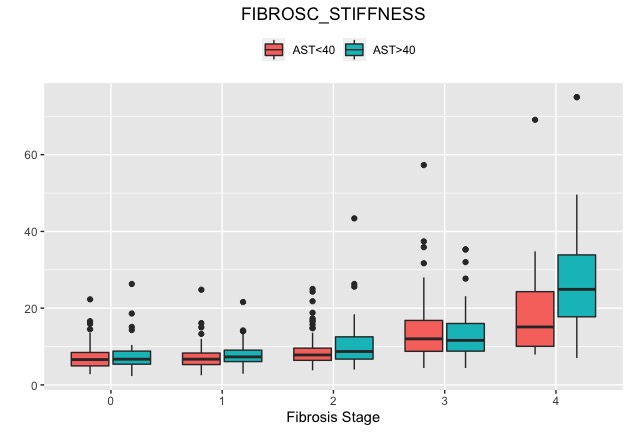


**1f)**

**
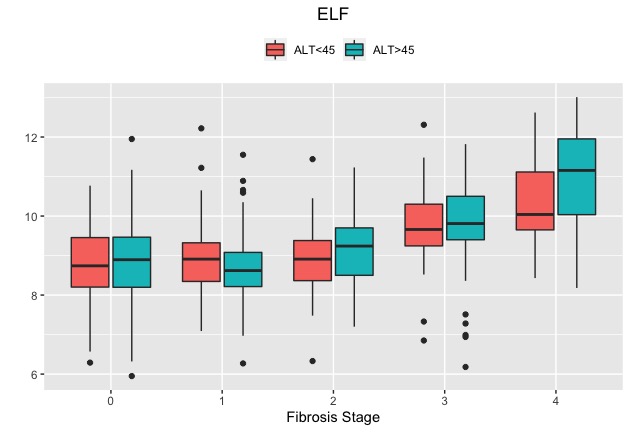
**

**
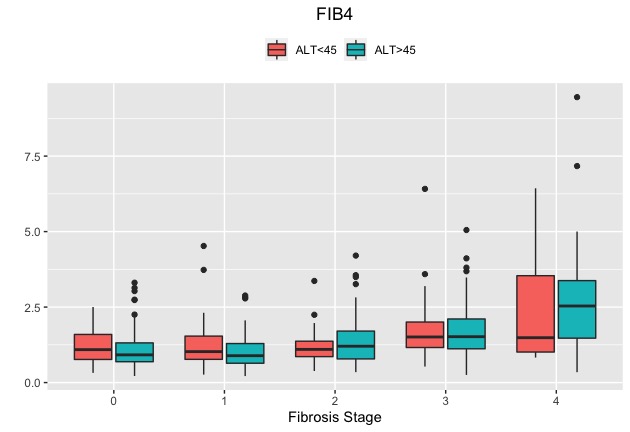
**

**
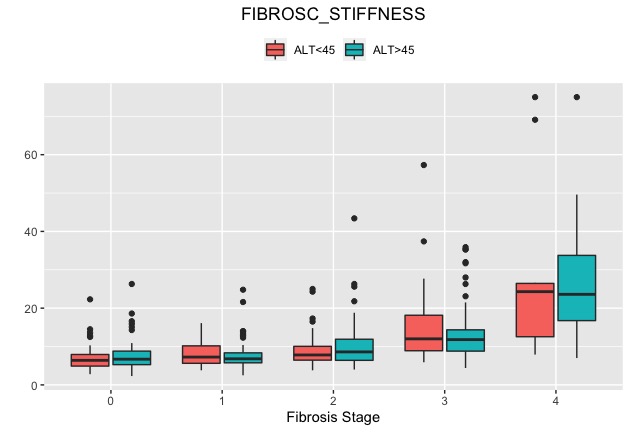
**
